# Influence of Skull Fracture on Traumatic Brain Injury Risk Induced by Blunt Impact

**DOI:** 10.3390/ijerph17072392

**Published:** 2020-04-01

**Authors:** Lihai Ren, Dangdang Wang, Xi Liu, Huili Yu, Chengyue Jiang, Yuanzhi Hu

**Affiliations:** 1 State Key Laboratory of Vehicle NVH and Safety Technology, China Automotive Engineering Reasearch Institute Co., Ltd. and Chongqing Chang’An Automobile Co., Ltd., Chongqing 401122, China; lihai.ren@cqut.edu.cn (L.R.); yuanzhihu@cqut.edu.cn (Y.H.); 2 Key Laboratory of Advanced Manufacturing Technology for Automobile Parts, Ministry of Education, Chongqing University of Technology, Chongqing 400054, China; wangdangdang@2018.cqut.edu.cn (D.W.); jiangchengyue@cqut.edu.cn (C.J.); 3 Chang’An Automobile Co., Ltd., Chongqing 400023, China; yuhl@changan.com.cn

**Keywords:** blunt impact, head finite element model, skull fracture, traumatic brain injury

## Abstract

This study is aimed at investigating the influence of skull fractures on traumatic brain injury induced by blunt impact via numerous studies of head–ground impacts. First, finite element (FE) damage modeling was implemented in the skull of the Total HUman Model for Safety (THUMS), and the skull fracture prediction performance was validated against a head–ground impact experiment. Then, the original head model of the THUMS was assigned as the control model without skull element damage modeling. Eighteen (18) head–ground impact models were established using these two FE head models, with three head impact locations (frontal, parietal, and occipital regions) and three impact velocities (25, 35, and 45 km/h). The predicted maximum principal strain and cumulative strain damage measure of the brain tissue were employed to evaluate the effect of skull fracture on the cerebral contusion and diffuse brain injury risks, respectively. Simulation results showed that the skull fracture could reduce the risk of diffuse brain injury risk under medium and high velocities significantly, while it could increase the risk of brain contusion under high-impact velocity.

## 1. Introduction

Road traffic collisions (RTCs) have become one of the most severe worldwide public health problems, and approximately 5 million people are injured and 1.2 million die from RTCs every year [1]. In China, approximately 200,000 RTCs occur annually, which leads to more than 200,000 injuries every year [2]. One of the main causes of the casualty in RTCs is severe traumatic brain injury (TBI) caused by head blunt impacts [3,4,5].

Blunt-impact head injuries of RTCs mainly include skull fracture and TBI [6,7]. The patterns of skull fracture depending on the initial head impact location (or direction), which can be divided into three main groups: anterior, lateral, and posterior, that is, frontal, lateral–parietal, and occipital, respectively [8,9,10]. According to the distribution of injury, TBIs can be divided into focal brain injuries and diffuse brain injuries (DBIs) [11,12,13]. Focal brain injuries usually lead to loss of partial brain function with contusions, intracranial hematomas, and so on [4,14]. DBIs include mild concussion, moderate concussion with brief coma, and diffuse axonal injury with long-term coma or death [12,13]. As demonstrated by real-world RTC data, approximately 50% and 25.8% of patients suffered from skull fractures [14] and cerebral contusions [3], respectively, while DBIs were found in approximately 35% of severe-head-injury-related deaths [11].

Even if numerous studies have been conducted to investigate mechanisms of blunt-impact head injury, the effect of skull fracture on TBIs has not been clearly understood. However, there is a significant correlation between skull fractures and TBIs [3,4,14]. Partial impact energy could be absorbed during the skull fractures, which could possibly reduce the energy transferred to the brain tissue [15]. Based on an investigation of the relationship between skull fractures and TBIs from 500 RTC-related head injuries, Yavuz et al. [3] indicated that the presence of skull fractures could lower the incidence of TBIs, while TBI-related patients without skull fractures are more likely to die in traffic accidents than those with skull fractures based on an investigation of 54 cases with RTC-related head injuries by Carson et al. [14]. However, because of the complexity of traffic accident conditions, the physiological structure of the human head, and the pathology of brain injuries, the mechanism of the influence of skull fractures on blunt impact is still unclear.

Therefore, finite element (FE) head–ground impact models were established, referring to an existing head–ground impact experiment. The objective of this study is to investigate the influence of skull fractures on the intracranial strain response of brain tissue associated with the brain contusion and DBIs following the head–ground impacts.

## 2. Methods

### 2.1. Head FE Model

**Figure 1:**
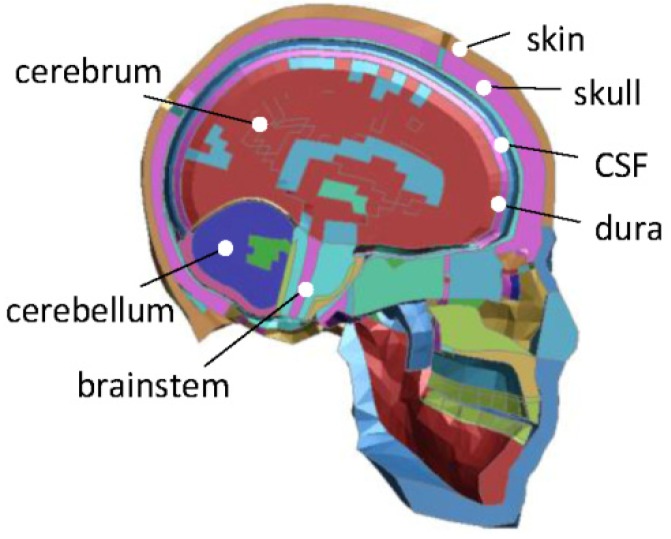
THUMS–head FE model.

**Figure 2:**
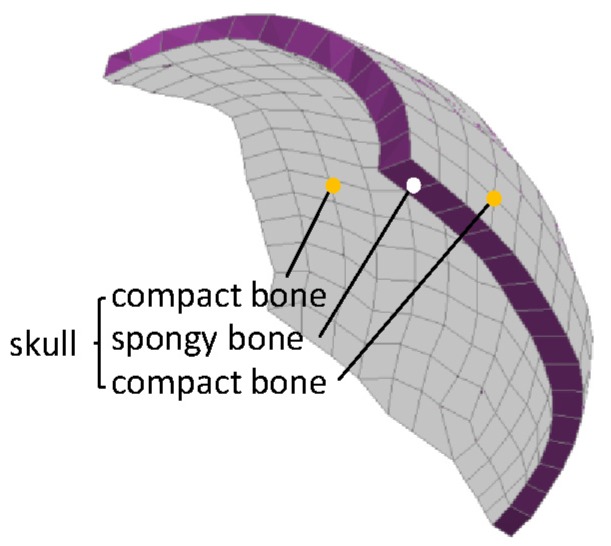
Three-layer structures of skull.

A 50th-percentile adult human head FE model (HFEM), extracted from the Total HUman Model for Safety (THMUS, Toyota Central R&D Laboratories, Nagakute, Japan; version 4.01) modeled by LS-DYNA software (version 971), was employed in this study. The most essential anatomical features of the human head are represented, including the scalp, skull, epidural, cerebrospinal fluid, cerebral white matter, cerebral gray matter, cerebellum white matter, cerebellum gray matter, brain stem, and brain chamber (Figure 1). The skull was modeled as a sandwich structure consisting of two layers of shell elements and one layer of solid elements, which presented the outer plastic material of the table of the compact bone and the inner elastic-viscoplastic material of the spongy bone, respectively (Figure 2). The Total HUman Model for Safety (THUMS)–head FE model has been extensively validated [16] and is widely used in the research field of the blunt impact-head injuries [17,18,19,20,21]. The detailed modeling method and parameters of the THUMS–head FE model are referenced in Toyota Motor Corporation documentation [16] and Kimpara et al. [22].

FE damage modeling was implemented in the skull of the original THUMS–head FE model in order to predict the possible skull fracture under head blunt impact. According to the published studies associated with skull fracture behavior and the following head impact loading conditions employed in this study [23,24,25], material properties of the skull element damage modeling were assigned as yield stresses of 95.88 and 4.794 MPa and plastic strain failure thresholds of 0.02 and 0.006, respectively, for compact bone and spongy bone.

### 2.2. Skull Fracture Prediction Performance Validation

**Figure 3:**
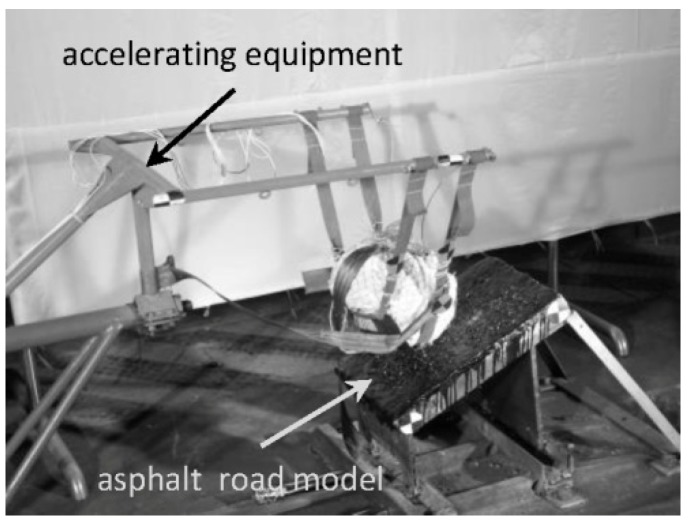
Head–ground impact experiment.

The skull fracture prediction performance of the improved THUMS–head FE model was evaluated via the reconstruction of an existing head–ground impact experiment carried out by Jiang et al. [26]. The original experimental equipment included an accelerator, asphalt road model, a high-speed camera, and an accelerating device (Figure 3). As demonstrated in the referenced study, the cadaver head was wrapped with white clothes and attached to the accelerating device by several seatbelts; the head was then accelerated to 44.1 km/h and impacted against the oblique ground at the occipital region; finally, the time history of the head acceleration at the foramen magnum region and the skull fracture were obtained through data acquisition system and CT scanner, respectively.

**Figure 4:**
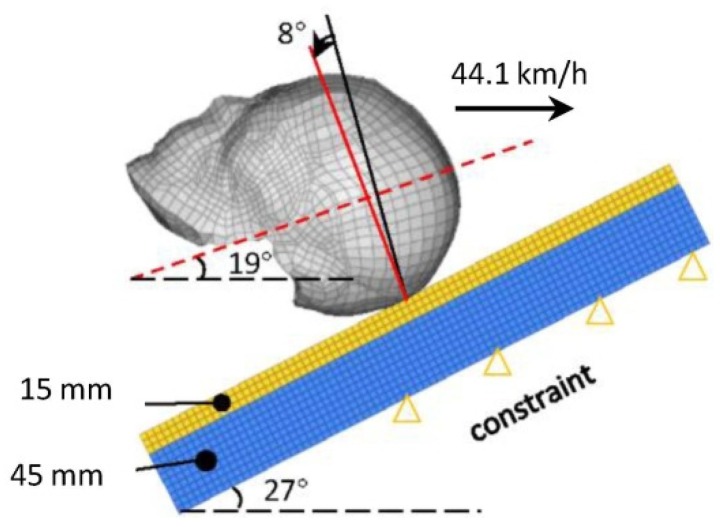
Setup of head-ground impact FE model. Black solid line is the parallel line of the vertical plane, and red solid line is that of the brain sagittal plane after rotating 8°. Red dotted line is located in the coronal plane of the head. The size of the ground is 500 × 500 × 60 mm.

A FE simulation model was established according to the referenced experimental data in LS-DYNA software, as shown in Figure 4. The ground with an asphalt surface (15 mm thick) and a concrete roadbed (45 mm thick) was modeled as an equivalent road using linear elastic solid elements [27] (Figure 4). The material parameters of the FE road model that have been applied in the pedestrian head–ground impact simulations were assigned [28] (Table 1). An initial velocity of 44.1 km/h was assigned to the head model, and the key word of *AUTOMATIC_CONTACT_SURFACE_TO_SURFACE was employed for the head–ground contact with a friction coefficient of 0.58 [29,30].

**Figure 5:**
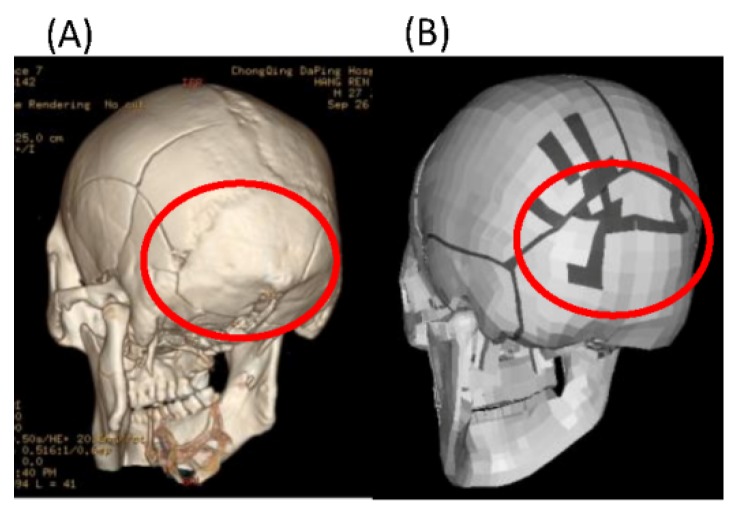
Comparison of skull fracture patterns between the (**A**) experiment and (**B**) simulation.

**Figure 6:**
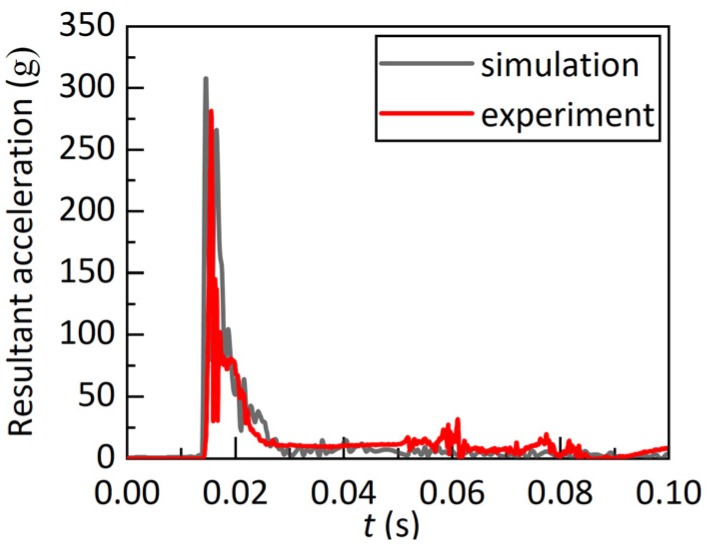
Comparison of head resultant acceleration between the experiment and simulation.

The predicted skull fracture and head acceleration at the foramen magnum are illustrated in Figure 5 and Figure 6, respectively. The skull fracture predicted at the head impact location (occipital region) correlated well with the corresponding skull fracture observed in the referenced experiment (Figure 5). As illustrated in Figure 6, the predicted time history of the resultant head acceleration was also well consistent with the referenced experimental data, with peak values of 307.79 and 281.32 g. Despite the missed prediction of the skull fracture at the temporoparietal region, the current head model indeed applied for the following simulation.

### 2.3. Head–Ground Impact Simulation Matrix

**Figure 7:**
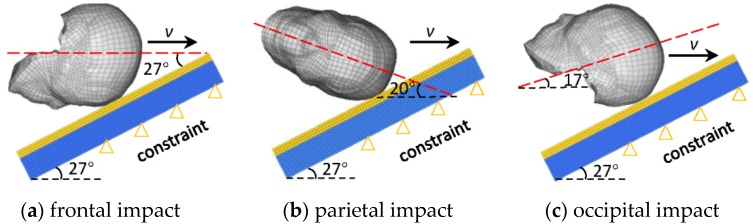
Illustrations of head–ground model for three impact locations. Red dotted line is located in the coronal plane of the head for (**a**) and (**c**) and in the mid-sagittal plane of the head for (**b**).

A matrix of head–ground impact simulation was performed using the current improved THUMS–head FE model and the original THUMS–head FE model for a quantitative comparison of the predicted intracranial dynamic responses under three head impact locations: frontal, lateral–parietal, and occipital regions. Here, the current improved THUMS–head FE model was assigned as the fracture model, while the original THUMS–head FE model was assigned as the non-fracture model. Three head impact velocities, 25, 35, and 45 km/h (low, medium, and high) were assigned to each model of three impact locations. Thus, a total of 18 simulations were performed. The detailed boundary conditions are illustrated in Figure 7.

To simulate the same risk of brain injuries in models of three impact locations at the same impact velocity, different impact angles of the head were designed. Here, the Brain Injury Criteria (BrIC) were calculated.

The BrIC was developed by Takhounts et al. [31], based on the mechanism of the head rotational motion for brain injuries. Furthermore, angular velocity is the only component of the BrIC. Therefore, angular velocities of the head model in three axes were extracted, and BrIC were calculated to assess the risk of brain injury. BrIC are expressed as follows:

(1) $$BrIC=\sqrt{{\left(\frac{\omega_{x}}{\omega_{xC}}\right)}^{2}+{\left(\frac{\omega_{y}}{\omega_{yC}}\right)}^{2}+{\left(\frac{\omega_{z}}{\omega_{zC}}\right)}^{2}}$$

where *ω_x_*, *ω_y_*, and *ω_z_* are maximum angular velocities about the *X*-, *Y*-, and *Z*-axes respectively; *ω_xC_*, *ω_yC_*, and *ω_zC_* are the critical angular velocities in their respective directions, which are 66.25, 56.45, and 42.87 rad/s [31]. The BrIC values at 50% risk of the Abbreviated Injury Scale (AIS) 2+, and 4+ brain injuries are 0.5 and 1, respectively [31].

### 2.4. Intracranial Dynamic Response and Data Analysis

#### 2.4.1. Intracranial Dynamic Responses

The maximum principal strain (MPS) and cumulative strain damage measure (CSDM) were calculated and assigned as the injury indexes for the proper prediction of cerebral contusion and diffuse brain injury, respectively. The MPS has been suggested as an efficient injury index of cerebral contusion under blunt impact [32,33]. In this study, 18 elements in a cube region in which the maximum strain response appeared in the brain impact region were selected for a calculation of average peak MPS. The CSDM has long been used as an essential injury index for the prediction of the DBI caused by brain tissue deformation [34,35], and is defined as the volume percentage of elements with the peak MPS exceeding a certain strain threshold for the target regions, as follows:

(2) $$CSDM=\frac{V\;\left(\varepsilon_{1}\geq \varepsilon_{c}\right)\;}{V}$$

where *ε_1_* is the elemental peak MPS and *ε_c_* the defined strain threshold. The white matter of the cerebrum and brainstem was selected as the target regions for the CSDM calculation, and the current strain threshold was set to 0.15 [34,35,36].

#### 2.4.2. Data Analysis

Differences in average peak MPS and CSDM between the fracture and non-fracture models were quantitatively analyzed by one-way analysis of variance (one-way ANOVA, Office Excel 2016). First, the MPS time history curves of the selected 18 elements were obtained from each simulation; then, one-way ANOVA was performed to evaluate the difference between the average peak MPS of the two head models under the same impact conditions. The difference between the two groups of CSDM predicted by the corresponding two head models was also evaluated by using the one-way ANOVA. The F-test was conducted to determine whether the skull fracture had a statistically meaningful effect on the average peak MPS and CSDM results: When the *p*-value is lower than 0.05, it can be said that a significant effect exists; otherwise, there is no such significant effect.

## 3. Results

### 3.1. Skull Fractures and Head Kinematic Responses

**Figure 8:**
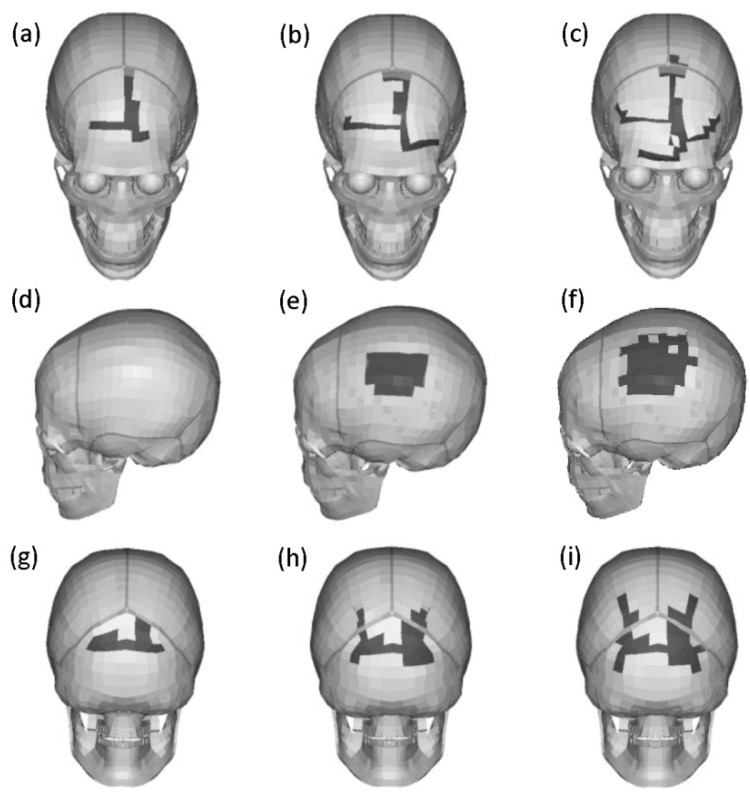
Fracture patterns of head fracture model for three locations under three impact velocities. (**a–c**) Frontal impact, (**d–f**) parietal impact, and (**g–i**) occipital impact; the impact velocities are 25, 35, 45 km/h from left to right, respectively; the skull fracture is represented in black.

Figure 8 shows that varying degrees of fracture are predicted in these impact regions with different head impact velocities. In general, there were distinct differences in fracture patterns in different skull regions. For 25 km/h, only small linear skull fractures were predicted in the frontal and occipital regions (Figure 8a,g, respectively); but no fracture was predicted in the parietal region (Figure 8d). Severe linear skull fractures and multiple skull fractures were predicted in the frontal and occipital regions at 35 and 45 km/h, respectively (Figure 8b,c,h,i). However, severely depressed skull fractures appeared in the parietal region with the head impact velocities of 35 and 45 km/h (Figure 8e,f).

**Figure 9:**
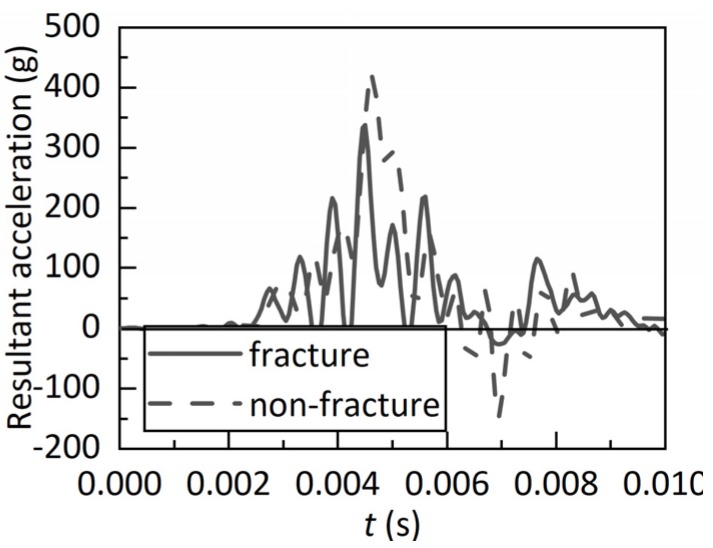
Comparison of resultant acceleration (filter of 1000 Hz) with parietal impact contrecoup side between fracture and non-fracture models at 35 km/h.

Figure 9 shows the comparison of the resultant acceleration (filter of 1000 Hz) with a parietal impact contrecoup side between fracture and non-fracture models at 35 km/h. The predicted resultant acceleration of the fracture model was 19.8% lower than the corresponding value of the non-fracture model.

**Figure 10:**
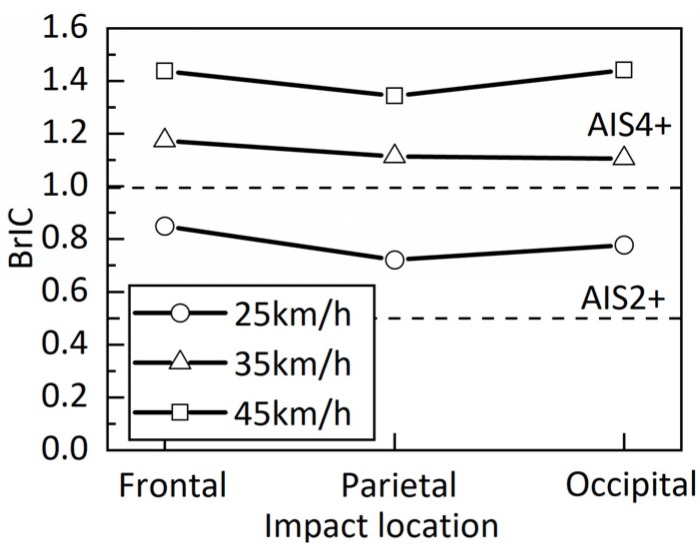
BrIC of the non-fracture model for three impacts at three impact velocities. Dotted lines are 50% risk of AIS 2+ and AIS 4+ brain injuries from the bottom up.

Figure 10 shows the BrIC of the non-fracture model for three impact locations under three impact velocities. The calculated average BrIC values of the three impacts at 25, 35, and 45 km/h were 0.78, 1.13, and 1.41, respectively. According to the BrIC risk curves of brain injuries [29], the AIS 2+ injuries could be realized in all simulations.

### 3.2. Strain Responses of Brain Tissue

**Figure 11:**
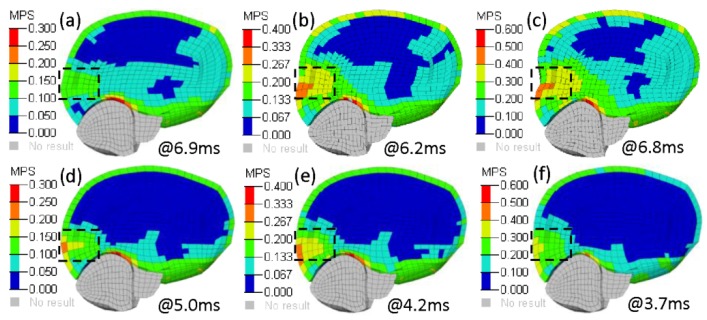
MPS contour for occipital impacts at three impact velocities. (**a–c**) Fracture model, (**d–f**) non-fracture model; impact velocities are 25, 35, and 45 km/h from left to right; dotted line box is at the peak MPS region of impact.

Figure 11 shows the MPS contour for occipital impacts at three impact velocities. At medium and high impact velocities, the brain of the fracture model exhibited severe extrusion deformation, which resulted in relatively higher MPS responses (Figure 11b,c).

**Figure 12:**
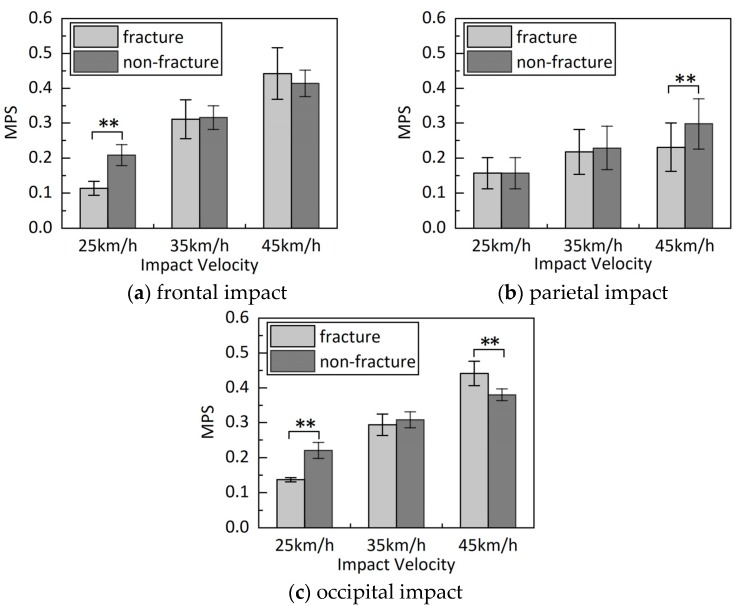
Average peak MPS from the impact region of three impact models under three impact velocities. Shown are the average and standard deviations of peak MPS response of 18 elements in the selected region. Asterisks on fracture and non-fracture models indicate the significant level of skull fracture effect on MPS peaks at each velocity; * denotes significant effect, that is, 0.01 < *p* ≤ 0.05; ** denotes extremely significant effect, that is, *p* ≤ 0.01. (**a**) frontal impact (**b**) parietal impact (**c**) occipital impact.

The average peak MPS values of the selected elements of the fracture model are lower than the corresponding values of the non-fracture model at 25 and 35 km/h, as illustrated in Figure 12. For 25 km/h, compared with MPS values of the non-fracture model, corresponding values of the fracture model were 45.7%, 0.3%, and 38.0% lower, for the frontal, parietal, and occipital impacts, respectively. Similarly, for 35 km/h, the MPS values of the fracture model were 1.5%, 4.5%, and 4.5% lower than the corresponding values of the non-fracture model, respectively. Inversely, the average peak MPS values of the fracture model were generally higher than the corresponding values of the non-fracture model at 45 km/h. For 45 km/h, compared with MPS values of the non-fracture model, corresponding values of the fracture model were 6.7%, −22.8%, and 16.1% higher for the frontal, parietal, and occipital impacts, respectively.

As shown in Figure 12, there was a significant effect of the skull fracture on average MPS values; except for the parietal impact at 25 km/h (*p* = 0.975), the *p*-values were less than 0.01 for frontal and occipital impacts. However, there was no significant effect of skull fractures on the average MPS values for all simulation at 35 km/h (*p* > 0.05). Inversely, significant effects were observed for all simulations at 45 km/h with *p*-values less than 0.01.

### 3.3. CSDM

**Figure 13:**
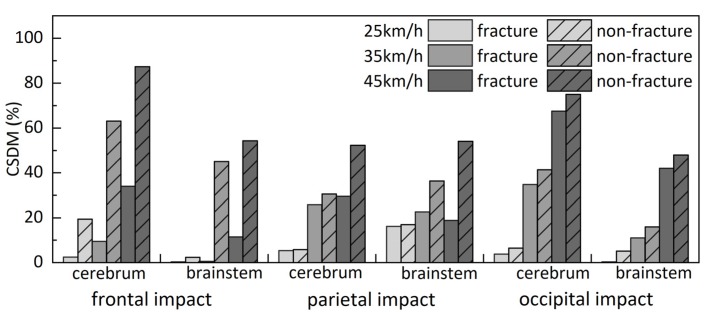
Comparison of cumulative strain damage measure (CSDM) between fracture and non-fracture models (strain threshold of 0.15).

**Figure 14:**
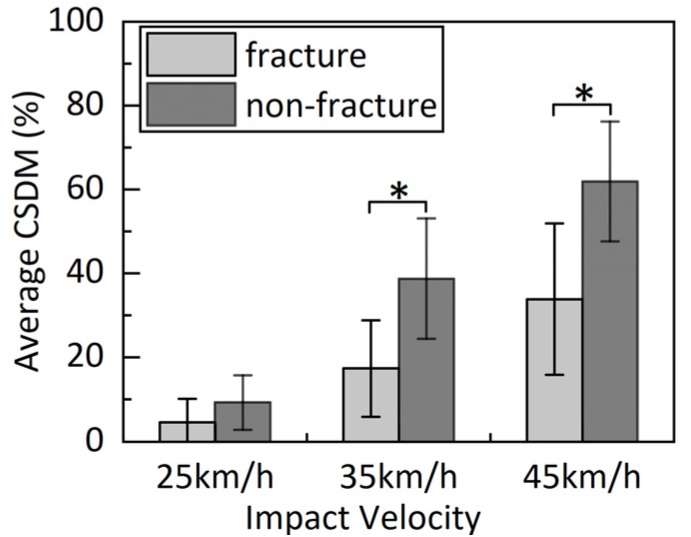
Comparison of average CSDM between head fracture and non-fracture models. Shown are average CSDM and standard deviation of the white matter of the cerebrum and brainstem for three impact velocities. Asterisks on fracture and non-fracture models indicate the significant level of skull fracture effect on CSDMs at each velocity; * denotes significant effect, that is, *p* ≤ 0.05.

CSDM values of the white matter of the cerebrum and brainstem for models of three impact locations are illustrated in Figure 13. CSDM values of the fracture model were lower than the corresponding values of the non-fracture model. The average CSDM values and standard deviation of three impact velocities are illustrated in Figure 14. Compared with CSDM values of the non-fracture model, corresponding values of the fracture model were decreased by an average of 49.3%, 55.0%, and 45.2% at 25, 35, and 45 km/h, respectively. In addition, significant differences could be observed for simulations at 35 km/h (*p* = 0.027) and 45 km/h (*p* = 0.022).

## 4. Discussion

The objective of this study is to investigate the influence of the skull fracture on TBIs under head-ground impacts. A total of 18 head–ground impact simulations were conducted by applying the newly improved THUMS–head FE model with skull element damage modeling and the original THUMS–head FE model, while three head impact locations (frontal, parietal, and occipital regions) and three impact velocities (25, 35, and 45 km/h) were employed. As the impact location was varied, the BrIC values of three impact models were calculated to assess whether the risk of brain injuries was consistent at the same velocity. A 50% risk of brain injury predicted by the three impact models was generally consistent at the same velocity (Figure 10). Therefore, three impact models were utilized to investigate the influence of skull fractures on brain injuries, which are suitable for this study.

As illustrated in Figure 8, different head impact locations could result in different skull fracture patterns [3,37,38,39]. Linear fractures in frontal [37] and occipital regions and depressed fractures in the parietal region were found at higher velocities [3], and fracture patterns were observed to be consistent with those commonly reported in the literature. In addition, the predicted resultant acceleration of the fracture model was lower than the corresponding value of the non-fracture model (Figure 9), suggesting that a certain amount of impact energy could be absorbed during the skull fracture [15,39].

The power of the MPS for the prediction of cerebral contusion has been confirmed in experimental studies [33]. Therefore, the MPS of two models at the impact region of the cerebrum were compared with the analysis of the influence of skull fractures on contusions in this study. For low impact velocity, the MPS values of the fracture model were significantly lower than the corresponding values of the non-fracture model, except for the parietal impact. When a small linear skull fracture occurred, the skull could still play a protective role regarding the brain tissue, and also a certain amount of impact energy could be absorbed, which could reduce the energy transferred to the brain tissue. However, for high-impact velocity, the MPS values of the fracture model were distinctly higher than the corresponding values of the non-fracture model, except for the parietal impact. The higher MPS values at the region of impact in the presence of the skull fracture model are likely induced by direct impression (Figure 11) [40]. Compared with the cerebral deformations of the frontal and occipital fracture models, a probable reason for the relatively low MPS obtained in the parietal impact of the skull fracture model at high head impact velocity was that the cerebrum suffered a relatively small deformation at the region of impact.

For all of these impact conditions, the predicted CSDM values of fracture models were lower than the corresponding values of non-fracture models. CSDM values could be reduced significantly with the appearance of skull fractures, especially for frontal and parietal impacts. Even though the appearance of a skull fracture has no significant effect on the CSDM values at low head impact velocity, the average CSDM values of the fracture models are generally relatively lower than corresponding values predicted by non-fracture models, with an average reduction of 49.3%, and the results observed were consistent with those reported in Carson et al. [14] study. As previously discussed, a certain amount of energy was absorbed during the skull fracture [15,39], while still being able to protect the brain. Therefore, we could deduce that the presence of skull fractures can reduce the injury risk of DBIs.

Owing to the lack of data on head trauma in traffic accidents, the impact of skull fracture on the intracranial response cannot be quantified for validity, which needs further improvement.

## 5. Conclusions

Simulation of head–ground impact models of three locations predicted MPS and CSDM values that had an effect between the fracture and non-fracture models. The influences of skull fracture on cerebral contusion and diffuse brain injury were investigated.

The results show that MPS values could be reduced significantly after skull fracture at low head impact velocity, which presents a lower risk of cerebral contusion. Conversely, the risk of cerebral contusion rises significantly after skull fracture at higher head impact velocity.

CSDM values are employed to evaluate the risk of DBIs. Overall, the predicted average values of these injury indices of the skull fracture model are lower than the corresponding values of the non-fracture model. Therefore, it was found that skull fracture significantly reduces the risk of DBIs per results obtained using current head–ground impact models.

## Figures and Tables

**Table 1:** Material properties of asphalt concrete road FE model.

Structure	Density/ (kg/m^3^)	Elasticity Modulus (MPa)	Poisson’s Ratio	Material Model
asphalt	1600	5400	0.35	linear elastic
roadbed	1600	9300	0.35	linear elastic
